# Exploring Strategies to Mitigate the Lightness Effect on the Prediction of Soybean Oil Content in Blends of Olive and Avocado Oil Using Smartphone Digital Image Colorimetry

**DOI:** 10.3390/foods12183436

**Published:** 2023-09-15

**Authors:** Isabella Marques de Carvalho, Yhan da Silva Mutz, Amanda Cristina Gomes Machado, Amanda Aparecida de Lima Santos, Elisângela Jaqueline Magalhães, Cleiton Antônio Nunes

**Affiliations:** 1 Department of Chemistry, Federal University of Lavras, P.O. Box 3037, Lavras 37203-202, MG, Brazil; 2 Department of Food Science, Federal University of Lavras, P.O. Box 3037, Lavras 37203-202, MG, Brazil; 3 Department of Engineering, Federal University of Lavras, P.O. Box 3037, Lavras 37203-202, MG, Brazilamandalsants@gmail.com (A.A.d.L.S.)

**Keywords:** chemometrics, prediction, authenticity, extra virgin olive oil, food fraud

## Abstract

Extra virgin olive oil (EVOO) and avocado oil (AVO) are recognized for their unique sensory characteristics and bioactive compounds. Declared blends with other vegetable oils are legal, but undeclared mixing is a common type of fraud that can affect product quality and commercialization. In this sense, this study explored strategies to mitigate the influence of lighting in order to make digital image colorimetry (DIC) using a smartphone more robust and reliable for predicting the soybean oil content in EVOO and AVO blends. Calibration models were obtained by multiple linear regression using the images’ RGB values. Corrections based on illuminance and white reference were evaluated to mitigate the lightness effect and improve the method’s robustness and generalization capability. Lastly, the prediction of the built model from data obtained using a distinct smartphone was assessed. The results showed models with good predictive capacities, R^2^ > 0.9. Generally, models solely based on GB values showed better predictive performances. The illuminance corrections and blank subtraction improved the predictions of EVOO and AVO samples, respectively, for image acquisition from distinct smartphones and lighting conditions as evaluated by external validation. It was concluded that adequate data preprocessing enables DIC using a smartphone to be a reliable method for analyzing oil blends, minimizing the effects of variability in lighting and imaging conditions and making it a potential technique for oil quality assurance.

## 1. Introduction

Edible oils are vital to our daily lives as food, in medicine, in cosmetic products, and for frying or cooking ingredients. Oils are also highly important in the human diet as they provide vitamins and fatty acids which are essential for development [1,2]. Among edible oils, extra-virgin olive oil (EVOO) is greatly appreciated among consumers for its pleasant sensory characteristics and renowned nutritional characteristics [3]. EVOO is processed exclusively by mechanical extraction without high temperatures or solvents, ensuring superior nutritional characteristics such as vitamin and antioxidant content, as well as its monounsaturated fatty acid profile [4]. Another edible oil that has recently become popular is avocado oil (AVO). AVO is often compared to EVOO in terms of nutritional quality due to its vitamin contents, bioactive compounds, and health claims related to its consumption [5,6].

Together with their distinct nutritional characteristics and popularity, EVOO and AVO are marketed at higher prices when compared to other edible oils. Therefore, these oils can be targeted for adulteration practices, mainly blending with lower-quality oils [2,3,7]. For that reason, developing methods and exploring techniques capable of detecting such frauds are required [8]. The studies in the scientific literature point to several techniques employed for fraud detection in EVOO, such as infrared spectroscopies (FTIR, MIR, and NIR) [9,10,11,12], chromatographic techniques [13,14], and nuclear magnetic resonance [15]. The studies on AVO fraud detection are also of a multi-equipment nature, including FTIR [2,6], NMR [16], RAMAN spectroscopy [17], and gas chromatography [18]. Although good detection was achieved in these cited studies, these analytical techniques generally have a high implementation or functioning cost and may require highly trained personnel. Therefore, more simple and low-cost approaches should be explored.

Among the alternatives, digital image colorimetry (DIC) is a promising candidate, and a recent focus of the scientific community in studies regarding oil quality [19,20,21,22,23]. The technique involves extracting color variables in a pre-defined color space (e.g., RGB), quantifying them, and constructing desired models [24]. Moreover, using DIC on smartphones further increases the possibility of in situ analysis. Indeed, the successful application of DIC on smartphones has been reported to classify different oils [25], aside from quantifying EVOO blending using smartphone videos [26]. However, some shortcomings of DIC on smartphones have been reported, especially those related to the effects of varying lighting conditions on the reproducibility of the results [24]. Therefore, this study aimed to explore strategies by which to mitigate the influence of lighting in order to make DIC using smartphones more robust and reliable for predicting the soybean oil content in EVOO and AVO blends.

## 2. Materials and Methods

### 2.1. Sample Preparation

Samples of extra virgin olive oil (EVOO) and avocado oil (AVO) were obtained from the Agricultural Research Company of Minas Gerais (EPAMIG), and a commercial brand of refined soybean oil (SO) was acquired at a local market. Two mixtures were prepared from the oils: extra virgin olive oil and refined soybean oil (EVOO-SO) and avocado oil and refined soybean oil (AVO-SO). For each mixture, the ratio (*w*/*w*) of one of the oils ranged from 0 to 1 (0 to 100%), with a 0.025 (2.5%) interval between points, resulting in a total of 41 proportions for each mixture. All samples were analyzed up to 3 days after the preparation.

### 2.2. Digital Image Acquisition

For image acquisition, 5 mL of each sample was placed in a 20 mL glass test tube supported on a tube rack and kept 15 cm from the smartphone camera. The samples were analyzed at ambient temperature (23 °C, controlled by the room climatization). Further, for standardization, a sheet of white bond paper was used as a background, supported on the back of the test tube (in relation to the camera). Two distinct smartphone models, the Moto G2 (8-megapixel camera, 3264 × 2448 pixels, F2.0 aperture) and the Samsung Galaxy S6 Edge (16-megapixel camera, 4640 × 3480 pixels, F1.9 aperture), were utilized to capture the digital images. The image-collecting technique was carried out in two rooms, one with natural illumination and the other with artificial lighting, to promote variation in lighting conditions. In the natural light setting, the room had two windows; the samples were placed on a counter 2 m from one of the windows, perpendicular to the windows through which daylight entered. As for the artificial light, the samples were analyzed in a square room without windows illuminated by four 40 W 6500 k tubular fluorescent lamps on the ceiling. The samples were placed on the bench on one side of the room. The images were represented by the average RGB values of the region of interest, centered between the base of the test tube and the sample, and obtained using the “Color Capture & Identifier” Android application.

### 2.3. Data Preprocessing

Since different analytical conditions can interfere with the model’s performance, as explicitly assessed in this study, the smartphone models and types, as well as data corrections, were tested to determine whether there was a method of preprocessing capable of enhancing the predictive quality of the built primary models when used to predict secondary (external) datasets. Two preprocessing methods were evaluated, one based on illuminance and the other on a white reference.

#### 2.3.1. Correction by Illuminance

Illuminance refers to the luminous flux incident on a surface per unit area. It can be considered the perceived brightness of visible light and is used to measure the intensity of light in the environment [27]. An illuminance value can be estimated as the Y component of the CIE tristimulus values (*XYZ*) [28].

In this work, a theoretical illuminance value (*Y_blank_*) was approximated from the RGB values [28] (*R_blank_*, *G_blank_*, and *B_blank_*) obtained for each sample from the empty portions of the test tubes directly above the oil samples:

*Y_blank_* = −0.32466 × *R_black_* + 1.57837 × *G_blank_* − 0.73191 × *B_blank_* (1)

The *Y_blank_* values were then used to generate corrected RGB values of the samples, according to Equations (2)–(4). This correction was called “illuminance”.

*R_corrected_* = *R_sample_*/*Y_blank_* (2)

*G_corrected_* = *G_sample_*/*Y_blank_* (3)

*B_corrected_* = *B_ample_*/*Y_blank_* (4)

#### 2.3.2. Correction by White Reference

Two blanks were considered for corrections based on white references: the mean RGB values found for the empty parts of the test tubes of the 41 calibration samples used for the primary model (primary mean blank) and each sample blank in the secondary conditions. In both cases, the RGB values were obtained from the empty part of the test tube in a region of interest just above the sample level.

The first correction was based on subtracting the RGB of the sample from the difference between the sample’s blank and the average blank of the primary samples, according to Equations (5)–(7). This correction was called “blanks difference”.

*R_corrected_* = *R_sample_* − (*R_sample blank_* − *R_average primary blank_*) (5)

*G_corrected_* = *G_sample_* − (*G_sample blank_* − *G_average primary blank_*) (6)

*B_corrected_* = *B_sample_* − (*B_sample blank_* − *B_average primary blank_*) (7)

Another correction was based on the product of the RGB of the sample by the ratio between the average blank of the primary samples and the sample’s blank, according to Equations (8)–(10). This correction was called the “blanks ratio”.

*R_corrected_* = *R_sample_* × (*R_average primary blank_*/*R_sample blank_*) (8)

*G_corrected_* = *G_sample_* × (*G_average primary blank_*/*G_sample blank_*) (9)

*B_corrected_* = *B_sample_* × (*B_average primary blank_*/*B_sample blank_*) (10)

The corrected RGB values were then used as descriptors in predictive models for predicting oil proportions.

### 2.4. Calibration Models

Each image was decomposed into the three coordinates of the RGB system using the “Color Capture & Identifier” application to obtain the descriptor variables. Each image was represented by the average RGB values of the region of interest, a 30 × 30-pixel area centered between the base of the test tube and the sample level. In addition to RGB values, two-by-two combinations of the coordinates (RG, RB, and GB) were also tested as descriptor variables.

Data sets for each of the two mixtures were split into calibration sets containing 80% of the samples and test sets, with the remaining 20% for external validation. The RGB, RG, RB, and GB values were then calibrated, using multiple linear regression (MLR), against the corresponding proportions of one of the oils.

The root mean squared error (RMSE) and coefficient of determination (R^2^) were the measures used to examine the models’ performances in the calibration, cross-validation, and external validation processes (using the test set). The r^2^m parameter was also used in the external validation stage to determine whether the measured and predicted values were congruent aside from being correlated; r^2^m values below 0.5 were regarded as acceptable [29]. The parameter R^2^p was employed to calculate the statistical difference between the R^2^ of the calibration and the R^2^ achieved by the y-randomization test. A valid distinction between the calibration and y-randomization R^2^ was defined by values equal to or higher than 0.5 [29].

All statistical procedures were performed using Chemoface, version 1.64 [30].

### 2.5. Method Robustness Assessment

Each set of mixtures (EVOO-SO or AVO-SO) was analyzed under four different analytical conditions, varying smartphone models, and types of lighting, as shown in Table 1.

The dataset obtained in the SG-IA condition was adopted as the primary model. To assess the robustness and generalizability of the technique, that is, the impact of the type of device and lighting on the accuracy of the predictions, the data sets obtained in other analytical conditions were treated as secondary and used as inputs to assess the primary model’s predictive power.

## 3. Results and Discussion

DIC using smartphones can be more reliable and robust when lighting is standardized [24], but efforts to standardize lighting can make these methods less practical. Therefore, this study focuses on investigating strategies by which to minimize the influence of lighting in order to make DIC using a smartphone more reproducible for predicting the soybean oil content in EVOO and AVO blends.

The first study subject was the EVOO-SO mixtures. The model’s figures of performance and validation for all the distinct modeling and correction approaches are presented in Table 2. The models in question, shown in Table 2, were primary models, i.e., they were analyzed using the Galaxy S6 Edge (SG) device under artificial lighting (IA).

For the EVOO-SO mixtures (Table 2), all the models constructed from the combination of color coordinates with or without preprocessing predicted the mixture content with a good R^2^ (>0.90) and low RMSE, both in calibration and external validation. The exception was a slightly lower performance found for the RG models, as indicated by their indices. Moreover, all models also showed good performances in the y-randomization test. As shown in Table 2, all models, independent of the color predictor or data preprocessing, showed R^2^_p_ > 0.5, indicating a valid correlation between the descriptors and the proportions of oils, i.e., no random data adjustment was made. In addition, to strengthen the findings for the obtained calibration curves, besides the high R^2^ obtained by the external validation, the r^2^_m_ parameter for the models was higher than 0.5, indicating that the predicted concentrations were not only close, but congruent, with the experimental data [29].

As no drastic difference was measured between the different color descriptors or data corrections, it would be reasonable to indicate the most straightforward approach for this calibration task. For that reason, using the RGB-based model without correction was chosen as a suitable calibration model for predicting the soybean oil content in olive oil.

According to the respective elected model, it was possible to detect soybean oil in EVOO with a prediction error of 2% (Table 2). These results are comparable to those obtained from more complex analytical setups. Oussama et al. (2012) [31] obtained a model with an R^2^ of 0.99 and an RMSE of 0.41 in external validation, detecting up to 24% of sunflower and soybean oil in EVOO using FTIR coupled with PLS regression. Moreover, Tan et al. (2018) [32] found an R^2^ of 0.99 and an error of 2% for the external prediction of corn, soybean, and sunflower oils in EVOO using front-face fluorescence and visible spectroscopy.

The similar results between our approach and the use of more complex analytical setups indicate the promising use of portable, lower-cost approaches as alternatives to quality monitoring tasks. Corroborating our findings, digital images with chemometrics have been reported on the classification of EVOO from different brands and adulterated samples [25]. Additionally, smartphone videos have also been successfully employed in tandem with multivariate calibration to quantify the level of vegetal oil intentionally added to EVOO [26].

As for the AVO-SO mixtures, the model’s performance and validation indices are displayed in Table 3. Differently from the EVOO-SO, all color descriptors were found to achieve outstanding performances, as measured by external validation. No evidence of random data adjustment was found, as assessed by the Y-randomization test, i.e., R^2^_p_ > 0.5 for any descriptor with any data correction. Interestingly, a higher accordance between the calibration and external validation R^2^ was found for the RGB and RG descriptors. On the other hand, the calibration R^2^ for the RB and GB descriptors was below 0.9 (Table 3). AVO has a more intense color than soybean oil, which may influence the color changes resulting from the mixture and the constructed models. Nonetheless, considering the overall evaluation and the parsimony principle, the model based on RGB without preprocessing was elected as the best model. Furthermore, in addition to its measured high R^2^ and low RMSE, the results from the R^2^m index with values higher than 0.5 indicated good congruence between the measured and predicted values.

In general, data preprocessing had little influence on the model’s quality. However, it is essential to note that the models obtained without preprocessing had already performed well, making obtaining relevant improvements challenging. Moreover, as for EVOO, studies on classification or adulteration detection in avocado oil are mainly performed with more sophisticated analytical setups. Quiñones-Islas et al. (2013) assessed the detection of avocado oil blending with sunflower, canola, and soybean oils by mid-FTIR spectroscopy. The multivariate calibration models in the range of 2–50% of mixing presented an R^2^ of prediction between 0.97 and 0.99, with a standard error of prediction between 0.08 and 2.81 depending on the oil which was mixed [6]. Through another approach, high-resolution nuclear magnetic resonance (NMR) was successfully employed in order to discriminate between avocado oil and EVOO [16]. Moreover, the data fusion between the fatty acid profile obtained by gas-chromatography and UV-Vis spectra resulted in a 100% accurate classification of avocado oil among other edible oils [33]. Despite its potential, not many reports can be found in the scientific literature on the assessment of the quality parameters of avocado oil using DIC.

Perez-Calabuig et al. (2023) used optical images and artificial neural networks to achieve a 95% accuracy classification of avocado oil blended with a range of 1 to 15% refined olive oil [34]. A higher number of reports exist regarding the successful quantification of quality parameters in avocado oil using smartphone images, such as peroxide values [21], chlorophyll and carotenoids [22], and total sterols [20].

Our results using smartphone images indicate the great potential of digital images to quantify EVOO and avocado oil blending as potential counterfeit practices. Furthermore, it is worth noticing that the models were built and tested from a dataset in which all samples were analyzed under the same light and equipment (smartphone) conditions, the SG-IA, with sample colors being the main factor with expected variation. To further evaluate the robustness and generalization capacity of the previously obtained models, they were used to predict the oil proportions using the datasets obtained in secondary analytical conditions as inputs, i.e., varying light and the use of a smartphone (SM-IN, CB-LA, and SM-IA). Table 4 shows the prediction of EVOO-SO obtained by the primary model using different secondary acquisition settings. The results have been shown to vary greatly as a function of the distinct color descriptors and preprocessing (Table 4).

**Figure 1:**
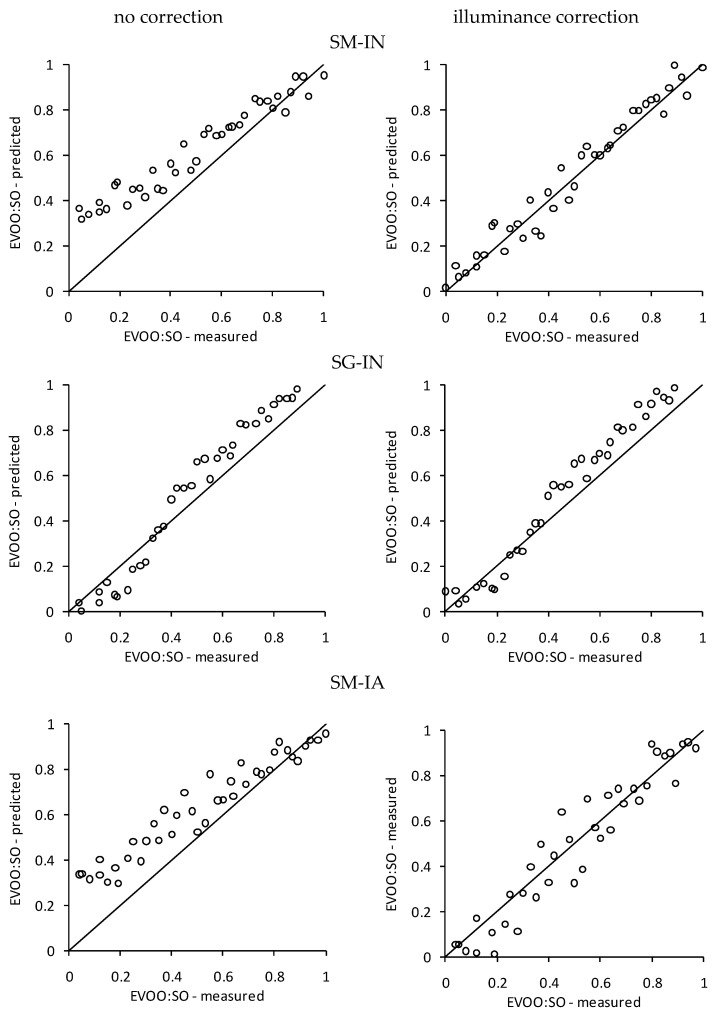
Comparison of observed and predicted extra virgin olive oil/soybean oil ratios using the GB-based model and different acquisition setups, both without preprocessing and with illuminance correction. SM: Smartophone Moto G2; SG: Smartphone Galaxy S6 Edge; IN: natural illumination; IA: artificial illumination.

Using secondary condition descriptors without preprocessing resulted in a considerably high RMSE and R^2^m < 0.5, indicating a lack of congruence between the predicted and measured values [29]. This finding relates to differences in the measured RGB values resulting from the smartphone camera and the type of lighting. These divergences were expected, as it is known that lightning and acquisition conditions are the main challenges of obtaining robustness and reliability whenusing DIC on a smartphone [24]. It is worth highlighting that the only models that achieved an R^2^m > 0.5 were the ones in which the secondary condition involved the SG smartphone, which was the one used in primary modeling, i.e., having a lower source of variation. Considering the preprocessing, an approach that led to an increase in model performance for all secondary acquisition conditions was the illuminance correction. It can be seen that all models, except the RG coordinate ones, led to a great improvement in model quality, achieving r^2^_m_ > 0.5, as well as a higher R^2^ and lower RMSE. This general increase in the models’ performances highlights the potential of this preprocessing to increase the generalization potential of the technique when applied to EVOO-SO mixtures. Further, Figure 1 shows the effect of the illuminance correction on the GB color predictors for the different secondary acquisition setups, indicating that the data became better fitted with the adjustment.

**Figure 2:**
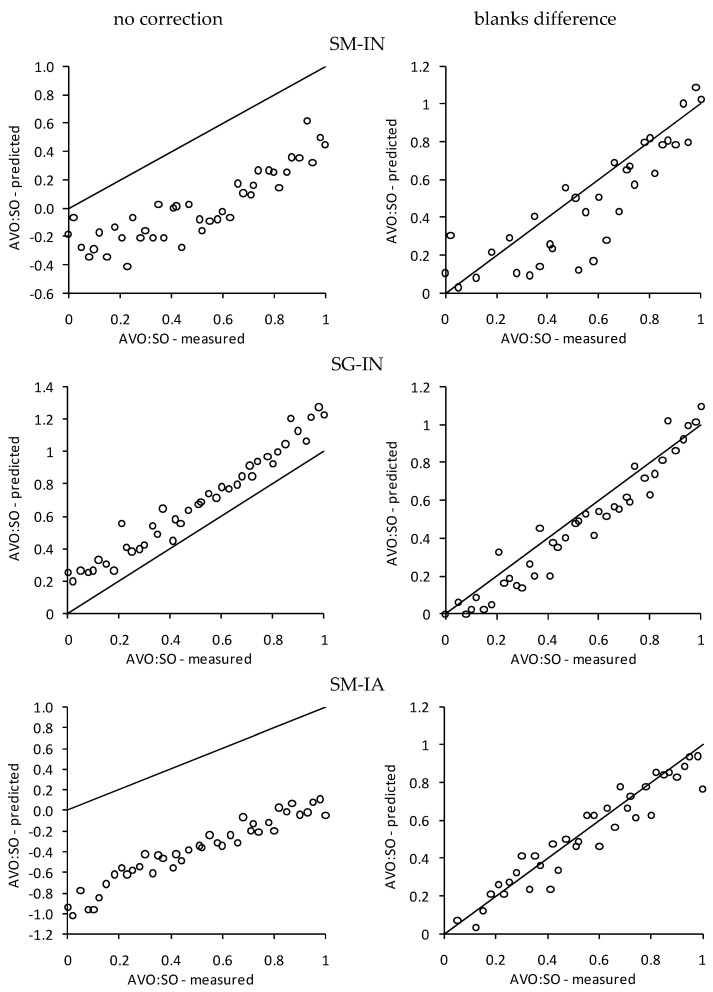
Comparison of observed and predicted avocado oil/soybean oil ratios using the GB-based model and different acquisition setups without preprocessing and with blanks difference correction. SM: Smartophone Moto G2; SG: Smartphone Galaxy S6 Edge; IN: Natural illumination; IA: Artificial illumination.

Table 5 shows the results of the primary model prediction of the avocado and soybean oil blend according to the secondary acquisition setups. Similar to the EVOO-SO results, the modeling without preprocessing led to a general lack of congruence between the observed and predicted values (r^2^_m_ < 0.5). The correction that predominantly increased this lack of congruence was the blanks difference. Looking specifically into the RB and GB models, a lowering in the RMSE was perceived, indicating an improvement in the model, even though the R^2^ did not significantly change. This model improvement can be further confirmed by the plot shown in Figure 2.

In general, the smallest errors and the largest R^2^ and R^2^m were obtained using the GB-based model, with illuminance correction in the EVOO-SO mixture and blanks difference for the SO-AB mixture. The RMSE values for this secondary data acquisition setup of 0.08 for EVOO-SO and 0.14 for AVO-SO were close to the values of 0.02 and 0.08 found for their respective best primary models. Moreover, the overall primary model performance for the GB descriptors also proved to be satisfactory, as assessed by their external validation. This equivalence between the prediction performance and distinct acquisition setups achieved with the preprocessing is of utmost importance, as the obtained RGB coordinates rely on the acquisition system [35]. Nonetheless, as the validation results were close to those of the primary models, which presented performances comparable to the more sophisticated setups, it can be stated that validation of external conditions was achieved via the preprocessing.

## 4. Conclusions

Using DIC via a smartphone proved to be a feasible tool for predicting soybean oil blends in extra virgin olive oil and avocado oil. The first evaluation of a controlled lightning condition and model comparison among a single smartphone indicated the technique’s potential to predict the blending with a great determination coefficient and a low prediction error. Furthermore, the use of the proposed illuminance correction and blank subtraction led to good blending prediction through RGB coordinates obtained using the secondary conditions, i.e., with a distinct smartphone and lightning. Therefore, the results indicate that adequate preprocessing techniques are potential tools to overcome the effects of lightning and distinct acquisition devices. Altogether, the results indicate the reliability and robustness of DIC on a smartphone as a potential quality assurance tool to predict oil blending practices. The results found in the present study c a base for standardization and further development of studies related to oil quality surveillance. Further research should focus on the effect of oil cultivars, origins, brand variability, and even blend storage to assess this technique’s applicability as a screening for fraud.

## Figures and Tables

**Table 1:** Experimental design with the analytical conditions for assessing the robustness of the technique.

Condition	Smartphone	Illumination
SM-IN	Moto G2	natural
SM-IA	Moto G2	artificial
SG-IN	Galaxy S6 Edge	natural
SG-IA	Galaxy S6 Edge	artificial

**Table 2:** Performance and validation parameters of the olive oil/soybean oil ratio primary prediction models.

RGB					
EVOO-SO		No Correction	Illuminance	Blanks Difference	Blanks Ratio
Calibration	RMSE	0.02	0.03	0.02	0.03
	R^2^	0.99	0.99	0.99	0.99
y-randomization	RMSE	0.28	0.28	0.27	0.28
	R^2^	0.12	0.11	0.14	0.12
	R^2^p	0.93	0.93	0.92	0.93
Cross-validation	RMSE	0.03	0.03	0.03	0.03
	R^2^	0.99	0.99	0.99	0.99
External validation	RMSE	0.04	0.04	0.04	0.04
	R^2^	0.98	0.98	0.98	0.99
	r^2^m	0.95	0.95	0.93	0.92
RG					
Calibration	RMSE	0.09	0.09	0.10	0.10
	R^2^	0.92	0.92	0.87	0.88
y-randomization	RMSE	0.29	0.29	0.29	0.29
	R^2^	0.05	0.04	0.04	0.04
	R^2^p	0.89	0.89	0.85	0.86
Cross-validation	RMSE	0.09	0.09	0.11	0.11
	R^2^	0.90	0.90	0.85	0.86
External validation	RMSE	0.11	0.11	0.14	0.14
	R^2^	0.88	0.89	0.77	0.77
	r^2^m	0.78	0.78	0.49	0.51
RB					
Calibration	RMSE	0.03	0.03	0.03	0.03
	R^2^	0.99	0.99	0.99	0.99
y-randomization	RMSE	0.29	0.29	0.29	0.29
	R^2^	0.04	0.04	0.05	0.06
	R^2^p	0.97	0.97	0.97	0.96
Cross-validation	RMSE	0.03	0.03	0.03	0.03
	R^2^	0.99	0.99	0.99	0.99
External validation	RMSE	0.04	0.04	0.04	0.04
	R^2^	0.98	0.98	0.98	0.98
	r^2^m	0.95	0.94	0.94	0.93
GB					
Calibration	RMSE	0.03	0.03	0.03	0.03
	R^2^	0.99	0.99	0.99	0.99
y-randomization	RMSE	0.29	0.29	0.28	0.29
	R^2^	0.04	0.04	0.08	0.04
	R^2^p	0.97	0.97	0.95	0.97
Cross-validation	RMSE	0.03	0.03	0.03	0.03
	R^2^	0.99	0.99	0.99	0.99
External validation	RMSE	0.04	0.04	0.05	0.04
	R^2^	0.98	0.98	0.98	0.98
	r^2^m	0.95	0.94	0.95	0.94

RMSE: Root mean squared error; R^2^: determination coefficient; R^2^_p_: determination coefficient of the difference between the y-randomized and original models; r^2^_m_: squared correlation coefficient between the predicted and observed values.

**Table 3:** Performance and validation parameters of the avocado oil/soybean oil ratio primary predicting models.

RGB					
AVO-SO		No Correction	Illuminance	Blanks Difference	Blanks Ratio
Calibration	RMSE	0.08	0.08	0.08	0.08
	R^2^	0.93	0.93	0.93	0.93
y-randomization	RMSE	0.28	0.29	0.29	0.28
	R^2^	0.12	0.06	0.08	0.10
	R^2^p	0.87	0.90	0.89	0.88
Cross-validation	RMSE	0.09	0.09	0.09	0.09
	R^2^	0.91	0.90	0.90	0.90
External validation	RMSE	0.06	0.05	0.08	0.07
	R^2^	0.96	0.97	0.94	0.95
	r^2^m	0.95	0.96	0.85	0.88
RG					
Calibration	RMSE	0.09	0.09	0.09	0.09
	R^2^	0.91	0.91	0.92	0.91
y-randomization	RMSE	0.29	0.29	0.29	0.28
	R^2^	0.04	0.05	0.07	0.11
	R^2^p	0.89	0.88	0.88	0.86
Cross-validation	RMSE	0.11	0.11	0.10	0.10
	R^2^	0.88	0.87	0.89	0.89
External validation	RMSE	0.07	0.06	0.08	0.07
	R^2^	0.94	0.96	0.94	0.94
	r^2^m	0.87	0.85	0.82	0.83
RB					
Calibration	RMSE	0.14	0.13	0.13	0.13
	R^2^	0.78	0.81	0.82	0.82
y-randomization	RMSE	0.29	0.29	0.29	0.29
	R^2^	0.06	0.05	0.08	0.05
	R^2^p	0.76	0.79	0.78	0.79
Cross-validation	RMSE	0.16	0.14	0.14	0.14
	R^2^	0.73	0.77	0.78	0.78
External validation	RMSE	0.12	0.15	0.11	0.11
	R^2^	0.92	0.86	0.95	0.95
	r^2^m	0.43	0.37	0.52	0.51
GB					
Calibration	RMSE	0.11	0.10	0.10	0.10
	R^2^	0.87	0.88	0.89	0.89
y-randomization	RMSE	0.29	0.29	0.29	0.29
	R^2^	0.04	0.08	0.04	0.06
	R^2^p	0.85	0.84	0.87	0.86
Cross-validation	RMSE	0.12	0.11	0.11	0.11
	R^2^	0.84	0.86	0.87	0.87
External validation	RMSE	0.08	0.10	0.08	0.08
	R^2^	0.96	0.94	0.97	0.97
	r^2^m	0.71	0.65	0.72	0.72

RMSE: Root mean squared error; R^2^: determination coefficient; R^2^_p_: determination coefficient of the difference between the y-randomized and original models; r^2^_m_: squared correlation coefficient between the predicted and observed values.

**Table 4:** Performance parameters of the olive oil/soybean oil ratio prediction models using secondary acquiring conditions as predictors.

	EVOO-SO	No Correction			Illuminance			Blanks Difference			Blanks Ratio		
		RMSE	R^2^	r^2^_m_	RMSE	R^2^	r^2^_m_	RMSE	R^2^	r^2^_m_	RMSE	R^2^	r^2^_m_
RGB	SM-IN	0.17	0.92	0.4	0.08	0.95	0.86	0.17	0.72	0.5	1.83	0.02	0.02
	SG-IN	0.12	0.97	0.71	0.08	0.98	0.86	0.12	0.97	0.72	0.09	0.96	0.81
	SM-IA	0.19	0.94	0.3	0.11	0.93	0.78	0.14	0.93	0.44	0.18	0.93	0.63
RG	SM-IN	0.54	0.59	0.23	0.91	0.51	0	1.64	0.04	0.03	20.14	0	0
	SG-IN	0.77	0.96	0	0.68	0.96	0	0.52	0.96	0.17	0.46	0.96	0.24
	SM-IA	0.32	0.62	0.43	0.65	0.62	0.18	0.49	0.35	0.24	0.93	0.35	0
RB	SM-IN	0.18	0.93	0.33	0.07	0.96	0.91	0.15	0.9	0.48	0.12	0.91	0.77
	SG-IN	0.1	0.97	0.76	0.08	0.98	0.9	0.09	0.97	0.79	0.08	0.96	0.89
	SM-IA	0.2	0.94	0.27	0.1	0.93	0.81	0.17	0.94	0.32	0.12	0.95	0.75
GB	SM-IN	0.16	0.95	0.31	0.06	0.96	0.94	0.22	0.72	0.32	1.18	0.08	0.07
	SG-IN	0.09	0.97	0.89	0.09	0.98	0.95	0.07	0.98	0.87	0.08	0.97	0.97
	SM-IA	0.16	0.94	0.33	0.09	0.93	0.85	0.22	0.92	0.17	0.09	0.93	0.85

RMSE: Root mean squared error; R^2^: determination coefficient; R^2^_p_: determination coefficient of the difference between the y-randomized and original models; r^2^_m_: squared correlation coefficient between the predicted and observed values; SM: Smartophone Moto G2; SG: Smartphone Galaxy S6 Edge; IN: natural illumination; IA: artificial illumination.

**Table 5:** Performance parameters of avocado oil/soy oil ratio prediction models, using secondary acquiring conditions as predictors.

	AVO-SO	No Correction			Illuminance			Blanks Difference			Blanks Ratio		
		RMSE	R^2^	r^2^m	RMSE	R^2^	r^2^_m_	RMSE	R^2^	r^2^_m_	RMSE	R^2^	r^2^_m_
RGB	SM-IN	0.33	0.28	0.23	0.39	0.32	0.29	0.38	0.32	0.3	0.49	0.21	0.17
	SG-IN	0.34	0.95	0.38	0.24	0.96	0.56	0.25	0.94	0.52	0.19	0.94	0.63
	SM-IA	0.33	0.83	0.42	0.67	0.85	0.07	0.34	0.87	0.41	0.76	0.86	0
RG	SM-IN	0.32	0.41	0.38	0.33	0.54	0.45	0.31	0.45	0.43	0.41	0.39	0.31
	SG-IN	0.22	0.93	0.57	0.15	0.94	0.71	0.22	0.92	0.57	0.15	0.92	0.71
	SM-IA	0.38	0.87	0.37	0.53	0.9	0.17	0.22	0.9	0.61	0.62	0.89	0.09
RB	SM-IN	0.61	0.74	0	0.33	0.7	0.43	0.25	0.74	0.6	0.29	0.77	0.51
	SG-IN	0.41	0.91	0.14	0.2	0.82	0.48	0.11	0.9	0.77	0.14	0.9	0.6
	SM-IA	1.08	0.85	0	0.28	0.8	0.48	0.2	0.83	0.48	0.21	0.83	0.61
GB	SM-IN	0.51	0.77	0.2	0.3	0.75	0.46	0.22	0.74	0.58	0.28	0.77	0.48
	SG-IN	0.19	0.96	0.7	0.09	0.92	0.86	0.1	0.94	0.82	0.07	0.95	0.93
	SM-IA	0.89	0.93	0	0.41	0.91	0.3	0.09	0.92	0.87	0.38	0.93	0.34

RMSE: Root mean squared error; R^2^: determination coefficient; R^2^_p_: determination coefficient of the difference between the y-randomized and original models; r^2^_m_: squared correlation coefficient between the predicted and observed values; SM: Smartophone Moto G2; SG: Smartphone Galaxy S6 Edge; IN: natural illumination; IA: artificial illumination.

## Data Availability

The data presented in this study are available onrequest from the corresponding author.
